# Copper homeostasis and cuproptosis: molecular mechanisms and therapeutic opportunities

**DOI:** 10.1186/s43556-026-00491-8

**Published:** 2026-07-08

**Authors:** Hepu Chen, Zhigang Zhou, Yixuan Tu, Yuanyuan Yang, Xu Liu, Nakhim Phoeurm, Jian Tu

**Affiliations:** 1 College of Pharmacy, Guilin Medical University, Guilin, Guangxi 541199 China; 2 Guangxi Key Laboratory of Diabetic Systems Medicine, Guilin Medical University, Guilin, Guangxi 541199 China; 3 The Second Affiliated Hospital of Guilin Medical University, Guilin, Guangxi 541199 China; 4 The First Affiliated Hospital of Jinan University, Guangzhou, Guangdong 510632 China; 5 Guangxi Key Laboratory of Molecular Medicine for Liver Injury and Repair, The Affiliated Hospital of Guilin Medical University, Guilin, Guangxi 541001 China; 6 College of Basic Medicine, Guilin Medical University, Guilin, Guangxi 541199 China; 7 College of Clinical Medicine, Guilin Medical University, Guilin, Guangxi 541199 China

**Keywords:** Copper homeostasis, Cuproptosis, Molecular mechanisms, Therapeutic opportunities, Liver disease

## Abstract

Copper, as an essential trace element, plays a critical role in various physiological processes including cell metabolism, nerve development, and immune function. Copper ions are maintained within an optimal range through a complex regulatory system in cells and organisms, ensuring dynamic equilibrium to sustain normal physiological functions and prevent copper toxicity. When this copper homeostasis is disrupted either by copper deficiency or overload, a series of pathological changes may occur, particularly in the liver, the primary organ responsible for copper metabolism. Cuproptosis is a unique form of regulated cell death specifically induced by copper ions, which has been identified mechanistically distinct from apoptosis, pyroptosis, and ferroptosis in recent research. Cuproptosis is initiated by the direct binding of copper to lipoylated proteins in the tricarboxylic acid (TCA) cycle, which leads to the aggregation of abnormal proteins, the loss of Fe-S clusters, and mitochondrial proteotoxic stress. Key regulators like the reductase FDX1 and the lipoyltransferase LIPT1 define this novel metabolic cell death pathway. As the central organ for copper metabolism, the liver is a primary site for copper homeostasis disruption and cuproptosis. Dysregulated copper metabolism and activated cuproptosis have been implicated in a spectrum of liver diseases, including Wilson's disease, metabolic dysfunction-associated steatotic liver disease (MASLD), alcohol-associated liver disease (ALD), and hepatocellular carcinoma (HCC). These findings provide profound insights into hepatic pathogenesis and reveal new therapeutic targets. This review summarizes the regulatory mechanisms of copper homeostasis, the related signaling pathways of cuproptosis, and the distinct mechanisms in various liver diseases. Furthermore, it highlights emerging therapeutic opportunities targeting copper ions to provide novel insights to explore and treat liver diseases.

## Introduction

As an essential micronutrient, copper is present in two distinct oxidation states: the oxidized (Cu^2^⁺) and the reduced (Cu⁺) [1]. Copper homeostasis is maintained by a delicate balance of intake, transport, and excretion, essential for normal growth and development in mammals. In humans, the maintenance of systemic copper homeostasis is a tightly regulated process involving intestinal absorption, systemic transport, hepatic storage, and biliary excretion, and coordination with utilization and excretion functions in other organs [2–4]. Copper ions are central to this process; by virtue of their redox properties, they act as catalytic cofactors, orchestrating a sequence of events through complex interactions with various intracellular enzymes and proteins [5].

Dysregulation of copper metabolism plays a dual role in both the pathogenesis of various diseases and the progression of tumors. On the one hand, copper is predominantly stored in the liver. So, any dysfunction of the liver can adversely affect copper storage, like excessive accumulation or impaired excretion, which will disrupt copper homeostasis in turn [6, 7]. However, this equilibrium disruption may result in copper accumulation, oxidative stress, gene expression alteration, and protein production inhibition [8]. On the other hand, copper participates in the process of cuproptosis, which has been identified as a significant form of cell death. The concept of cuproptosis was first proposed by Tsvetkov et al. [9], challenging traditional views of regulated cell death (RCD), marking a new milestone in the study of copper-induced cell death mechanisms.

Currently, there is growing interest in understanding how copper functions in vivo and how copper dysregulation relates to diseases. Consequently, elucidating its molecular mechanisms and evaluating its therapeutic potential have become critical issues requiring urgent exploration in this field. This review provides a systematic overview of copper's role in cancer. Firstly, we will discuss how to regulate copper to maintain dynamic equilibrium, the deep correlation between copper homeostasis and cuproptosis, the molecular mechanisms of cuproptosis, and its role and underlying mechanisms in liver diseases. Based on current emerging strategies targeting copper for disease treatment, we will explore how copper can both promote and suppress cancer. Subsequently, we review significant breakthroughs in this research field, including key molecules involved, and examine how copper signaling influences cancer progression. Furthermore, this review discusses novel therapeutic approaches targeting copper, with a focus on the importance of related genes in these treatments.

## Systemic and cellular copper homeostasis

Copper is an essential redox-active transition metal. As a catalytic cofactor for numerous cuproenzymes, it supports fundamental processes, including mitochondrial oxidative phosphorylation, antioxidant defense, connective tissue maturation, and neuropeptide activation [3, 4]. However, its potent redox capacity also renders copper a potential source of cytotoxic reactive oxygen species, necessitating stringent regulatory mechanisms at both systemic and cellular levels [5]. To manage this dual nature, organisms have evolved a sophisticated proteostatic network comprising transporters, chaperones, and storage proteins that collectively ensure precise copper delivery, utilization, and excretion. This tightly orchestrated system maintains copper homeostasis, thereby supporting enzymatic functions while mitigating redox-mediated damage [7].

Dysregulation of copper homeostasis is increasingly implicated in the pathogenesis of diverse human diseases, including metabolic, inflammatory, and autoimmune disorders. Notably, aberrant copper metabolism has emerged as a hallmark of cancer, influencing multiple facets of tumorigenesis—from proliferation and angiogenesis to metastasis and drug resistance [9]. The recognition of copper-dependent cell death (cuproptosis) has further unveiled the profound connection between copper metabolism and cellular fate, opening new therapeutic avenues [10]. Consequently, targeting copper regulatory pathways represents a promising strategy for treating a spectrum of diseases, highlighting the critical need to elucidate the molecular logistics of copper handling.

In this context, the following sections detail the systemic and cellular mechanisms governing copper homeostasis, beginning with its absorption, systemic distribution, and culminating in the intricate intracellular chaperone network that directs this essential yet potentially toxic metal to its correct destinations.

### Systemic copper absorption and distribution

Maintaining systemic copper homeostasis is crucial, given the element's widespread presence in the human body (total content 100–200 mg) with varying organ-specific concentrations [10]. To this end, a daily copper intake of 0.8 to 2.4 mg is advised [11], primarily sourced from the diet via the absorption of copper-rich foods, including animal offal, seafood, and nuts [12].

Copper homeostasis in mammals is a tightly orchestrated process essential for maintaining physiological balance and preventing toxicity [13]. Systemic copper absorption initiates in the duodenum and jejunum, where dietary Cu^2^⁺ is first reduced to Cu⁺ by apical metalloreductases, including members of the STEAP family and duodenal cytochrome b (DCYTB) [2, 14]. The resulting Cu⁺ is then transported across the apical membrane predominantly via copper transporter 1 (CTR1; encoded by *SLC31A1*) [15, 16], and CTR1 functions as a homotrimeric, high-affinity Cu⁺-selective permease that facilitates copper import down a concentration gradient without relying on ATP hydrolysis or a secondary ion gradient. The selectivity filter of CTR1 features layers of highly conserved methionine residues (notably Met-150 and Met-154) that coordinate Cu⁺ with high affinity, enabling efficient copper acquisition at low luminal concentrations. Recent evidence further demonstrates that elevated intracellular copper induces rapid monomerization of trimeric CTR1 at Rab5⁺ endocytic sites before the complex undergoes endocytosis, providing an acute regulatory mechanism that rapidly halts copper uptake to prevent intracellular overload [14]. Divalent metal transporter 1 (DMT1; encoded by *SLC11A2*) is also expressed on the apical membrane of enterocytes and has been shown to transport Cu^2^⁺, thereby serving a complementary role in dietary copper uptake. However, DMT1 exhibits considerably lower affinity for copper compared with CTR1, and its primary physiological substrates are Fe^2^⁺ and other divalent metal cations; copper transport by DMT1 is therefore most relevant under conditions of elevated luminal copper or when CTR1 function is compromised [2]. This hierarchical transporter system—dominated by high-affinity CTR1 trimers and supported by lower-affinity DMT1—ensures both the precision of copper acquisition and the flexibility to accommodate fluctuations in dietary copper intake. Following entry into the enterocyte, copper is chaperoned by the antioxidant protein 1 (ATOX1) to the basolateral membrane, where it is exported into the portal circulation via the P-type ATPase, ATP7A [17]. In the bloodstream, copper is predominantly bound to ceruloplasmin (CP), with the remainder associated with albumin and histidine [18, 19], facilitating its transport to the liver—the central hub for copper distribution and storage. Within hepatocytes, copper can be sequestered by metallothioneins (MTs) for storage or exported back into the systemic circulation via ATP7B for delivery to extrahepatic tissues [20, 21]. Excess copper is effectively eliminated from the body primarily through biliary excretion, a process also dependent on ATP7B, thus completing the cycle of systemic copper homeostasis [22, 23]. This exquisite regulation ensures the provision of copper as a critical cofactor for numerous enzymes while safeguarding against its inherent redox toxicity.

### Regulation of copper homeostasis

Within the cell, copper homeostasis is tightly regulated by a sophisticated network of transporters and chaperones [24, 25], ensuring this essential but potentially toxic micronutrient is delivered to its correct intracellular destinations with high fidelity [26]. The journey of copper begins at the plasma membrane, where the high-affinity copper transporter (CTR1; encoded by *SLC31A1*) serves as the primary gateway for the uptake of reduced copper (Cu⁺). This process is facilitated by metalloreductases of the STEAP family, which maintains extracellular copper in its Cu⁺ state [26, 27]. Upon entry, copper ions do not exist freely in the cytosol but are immediately sequestered by a network of specific molecular chaperones, forming a kinetically labile pool that minimizes the generation of harmful reactive oxygen species.

**Fig. 1:**
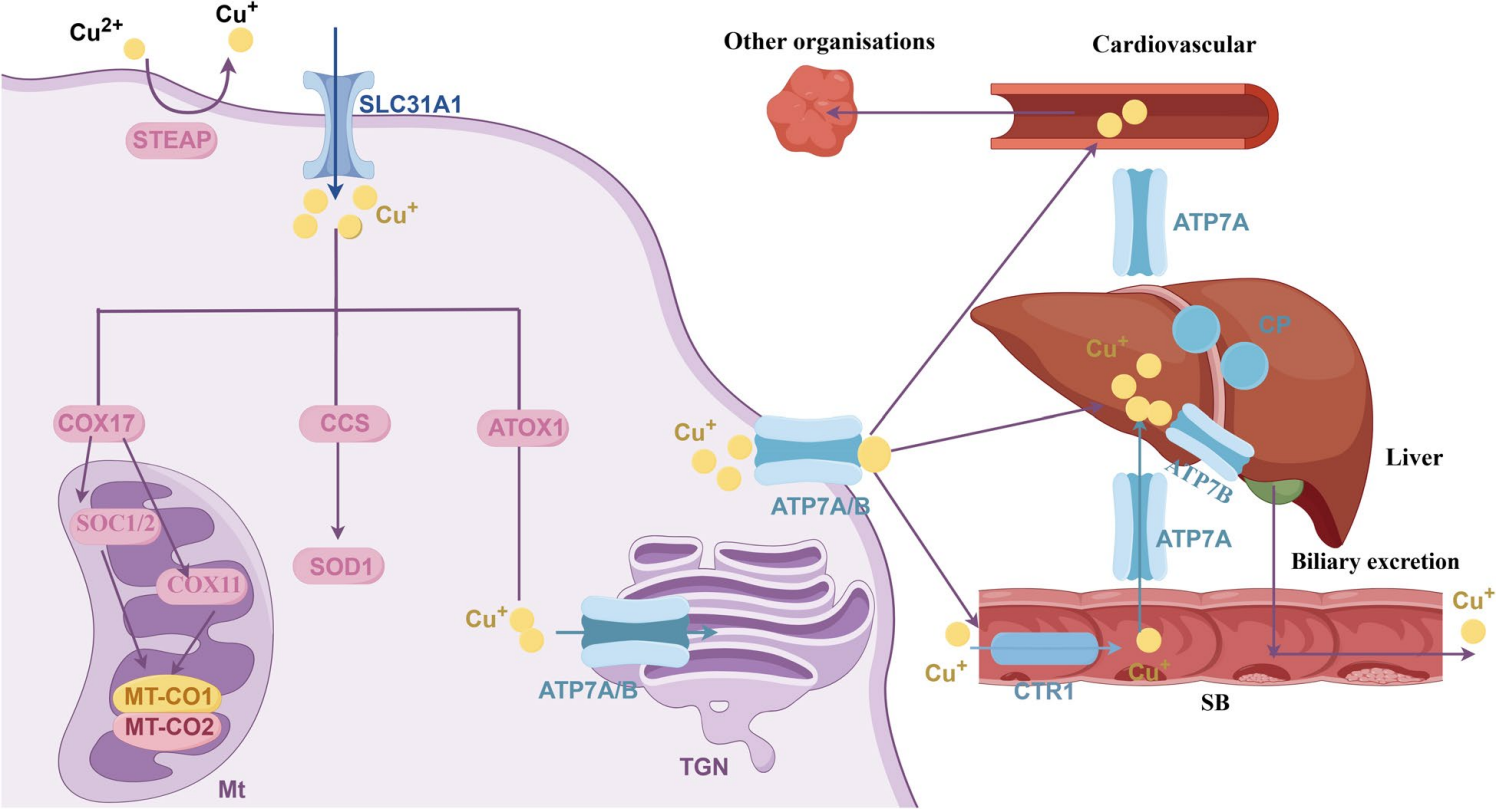
Schematic diagram of copper homeostasis and metabolism (by Figdraw) Copper ions enter cells primarily via the high-affinity copper transporter 1 (CTR1; encoded by *SLC31A1*). Upon reaching the cytoplasm, they are distributed to distinct subcellular compartments by a network of dedicated metallochaperones: antioxidant protein 1 (ATOX1) delivers copper to the trans-Golgi network (TGN); COX17 directs copper to the mitochondria, and the copper chaperone for superoxide dismutase (CCS) specifically mediates copper delivery to superoxide dismutase 1 (SOD1), enabling its enzymatic activation. At the TGN, the copper-transporting P-type ATPases ATP7A and ATP7B mediate copper loading into various cuproenzymes. Under conditions of copper excess, ATP7A relocates to the plasma membrane to promote cellular efflux, whereas ATP7B in hepatocytes facilitates biliary copper excretion. A fraction of copper is also taken up in the small bowel via CTR1. Systemic distribution of copper is subsequently achieved through its export into the bloodstream by ATP7A and ATP7B

This chaperone network functions as a precise delivery service, routing copper to distinct subcellular compartments. Cytosolic copper chaperone for superoxide dismutase (CCS) directly metallates superoxide dismutase 1 (SOD1) [28–30], activating this critical antioxidant enzyme in the cytosol and mitochondrial intermembrane space. Concurrently, the chaperone antioxidant 1 (ATOX1) shuttles copper to the trans-Golgi network, where it loads copper into the P-type ATPases ATP7A and ATP7B [17, 31]. Under normal conditions, these pumps incorporate copper into copper-enzymes like lysyl oxidases; during copper excess, they translocate to the plasma membrane to mediate efflux, constituting a key regulatory node. For mitochondrial utilization, the chaperone COX17 delivers copper to the intermembrane space, where it is transferred via SCO1/SCO2 and COX11 for the assembly of the cytochrome c oxidase (COX) complex [29, 32], essential for mitochondrial respiration. Recent research has further illuminated the central role of the reductase FDX1, which not only reduces Cu^2^⁺ to the more toxic Cu⁺ within mitochondria but also directly interacts with lipoic acid synthase (LIAS) [9, 33], thereby linking copper metabolism to the lipoylation of TCA cycle enzymes—a process at the very heart of cuproptosis (Fig. 1).

This elaborate trafficking system ensures that copper fulfills its vital roles as a catalytic cofactor while minimizing its cytotoxic potential. Dysregulation of this network, whether through aberrant expression of transporters like CTR1 or mutations in chaperones and efflux pumps, disrupts copper homeostasis. Such disruptions can contribute to oncogenesis by promoting copper-dependent proliferation (cuproplasia) or, conversely, can be therapeutically exploited to induce copper-dependent cell death (cuproptosis) in malignancies, particularly those reliant on mitochondrial metabolism like hepatocellular carcinoma. Therefore, a deep understanding of these molecular logistics is paramount for developing novel therapeutic strategies that target the copper axis in human diseases.

## Molecular mechanism of cuproptosis

### Direct binding of copper to lipoylated TCA cycle proteins

**Fig. 2:**
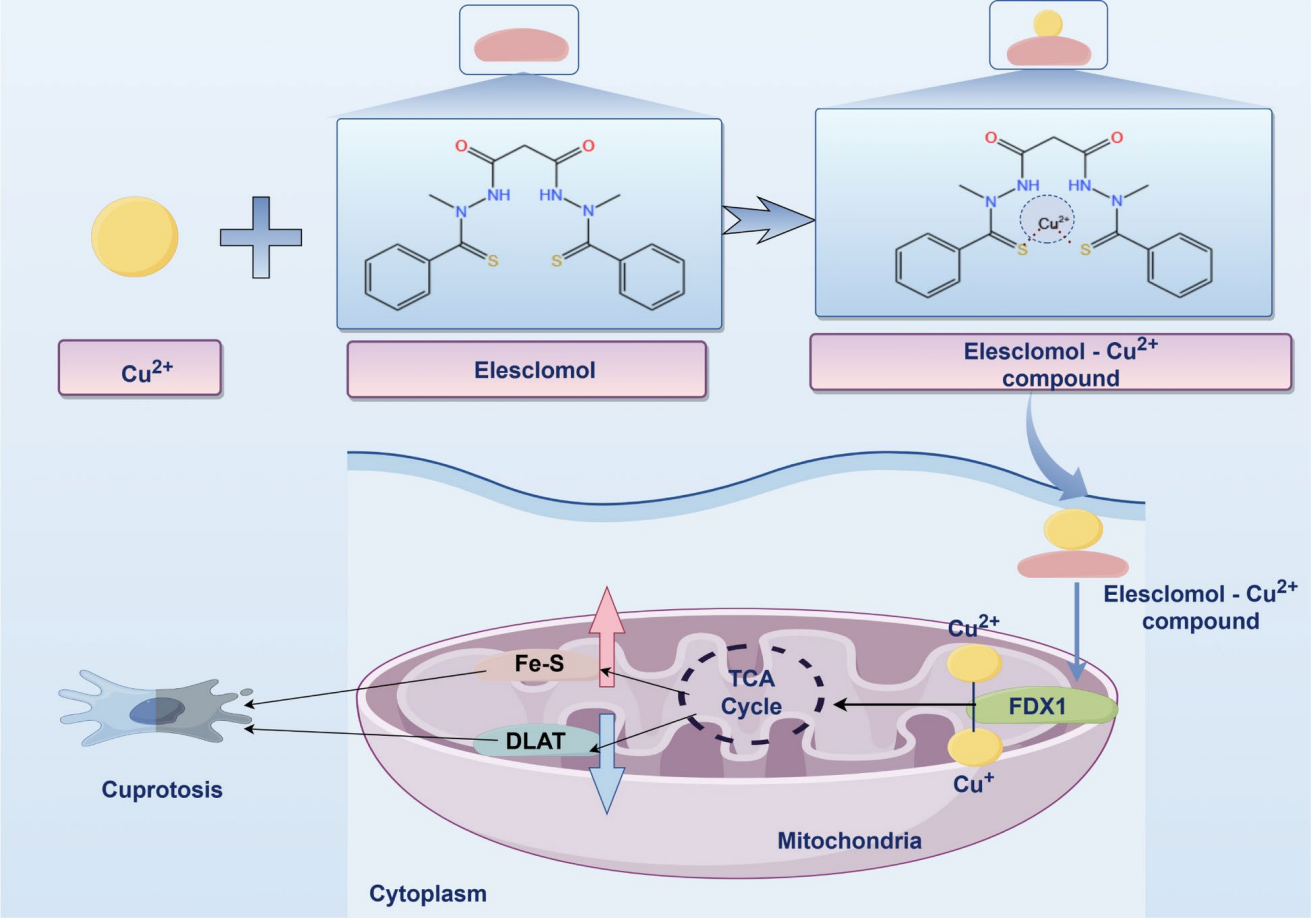
Schematic diagram of the cuproptosis mechanism (by Figdraw) Within the mitochondrial matrix, ferredoxin 1 (FDX1) performs two critical functions: it reduces Cu^2^⁺ to the more reactive cuprous ion (Cu⁺) and facilitates the lipoylation of key tricarboxylic acid (TCA) cycle enzymes through its interaction with lipoyl synthase (LIAS). The Cu⁺ generated then directly binds to these lipoylated substrates—most notably dihydrolipoamide S‑acetyltransferase (DLAT), a core E2 subunit of the pyruvate dehydrogenase (PDH) complex—triggering aberrant oligomerization and aggregation of lipoylated proteins. Concurrently, Cu⁺ overload destabilizes iron–sulfur (Fe–S) cluster‑containing proteins, thereby compromising respiratory chain integrity. The convergence of protein aggregation and Fe–S cluster loss overwhelms mitochondrial proteostasis, culminating in proteotoxic stress and cuproptotic cell death

Tsvetkov et al. elucidated cuproptosis as a novel, regulated cell death pathway, a landmark discovery that fundamentally reshapes our understanding of copper-induced cytotoxicity [9] by delineating a novel, regulated cell death pathway termed cuproptosis. Distinct from all other known forms of programmed cell death such as apoptosis, ferroptosis, and necroptosis [34, 35], cuproptosis is mechanistically defined by the direct binding of copper to lipoylated components of the tricarboxylic acid (TCA) cycle, leading to proteotoxic stress and eventual cell death [36]. This pathway is intrinsically linked to mitochondrial metabolism, positioning cellular bioenergetics as a key determinant of sensitivity to copper toxicity [37] (Fig. 2).

The execution of cuproptosis is intimately linked to the mitochondrial protein ferredoxin 1 (FDX1) [38, 39], which fulfills two indispensable biochemical functions. First, FDX1 catalyzes the single-electron reduction of cytotoxic Cu^2^⁺ to the more reactive Cu⁺ within the mitochondrial matrix, thereby generating the bioactive copper species required for downstream targeting. Second, FDX1 serves as an upstream regulator of mitochondrial protein lipoylation: through direct physical interaction with lipoic acid synthase (LIAS) [40], it promotes the covalent conjugation of lipoic acid moieties to conserved lysine residues on key TCA cycle enzymes, including dihydrolipoamide S-acetyltransferase (DLAT), a core E2 subunit of the pyruvate dehydrogenase (PDH) complex [33]. This FDX1-dependent lipoylation establishes the molecular substrate upon which copper exerts its cytotoxic effects, providing the mechanistic foundation for cuproptosis execution.

The reduced copper (Cu⁺) directly and specifically binds to the lipoylated domains of several TCA cycle enzymes. A primary target is dihydrolipoamide S-acetyltransferase (DLAT) [41], a core component of the pyruvate dehydrogenase (PDH) complex. The binding of copper induces aberrant oligomerization and aggregation of these lipoylated proteins [9, 42–46]. This aggregation overwhelms the mitochondrial protein folding capacity, triggering a severe proteotoxic stress response, which is evidenced by a marked upregulation of heat shock proteins (e.g., HSP70) [47, 48].

Alongside the aggregation of lipoylated proteins, copper toxicity concurrently destabilizes iron-sulfur (Fe-S) cluster proteins [9]. These clusters are vital cofactors for multiple enzymes involved in the electron transport chain and other metabolic processes. Their loss exacerbates mitochondrial dysfunction [49], contributing to the irreversible cascade toward cell death. The combination of lipoylated protein aggregation and Fe-S cluster depletion creates a lethal insult that the cell cannot resolve [9].

The reliance of cuproptosis on functional TCA cycle enzymes and mitochondrial respiration explains its unique metabolic dependency. Cancer cells or subpopulations with high oxidative phosphorylation (OXPHOS) activity [50], which express elevated levels of these lipoylated proteins, demonstrate heightened sensitivity to cuproptosis [49, 51, 52]. In contrast, cells reliant on glycolysis are relatively resistant. This vulnerability presents a therapeutic window for selectively targeting highly metabolic tumor cells [53].

In summary, the molecular mechanism of cuproptosis is centered on the FDX1-mediated reduction of copper and the subsequent direct binding of Cu⁺ to lipoylated TCA cycle proteins, notably DLAT. This interaction precipitates fatal protein aggregation and Fe-S cluster instability, culminating in a unique form of mitochondrial metabolism-dependent cell death. The elucidation of this pathway not only resolves long-standing questions regarding copper-specific cytotoxicity but also unveils a promising therapeutic strategy. Targeting the "FDX1-lipoylation-copper" axis holds immense potential for eradicating cancers characterized by enhanced mitochondrial respiration, stem-like properties, or therapy resistance.

### Consequences of copper-protein aggregation

At the molecular level, cuproptosis is initiated when excess copper accumulates in the mitochondrial matrix [54–56], where it undergoes a critical redox conversion catalyzed by ferredoxin 1 (FDX1). This iron-sulfur cluster-containing reductase specifically reduces Cu^2^⁺ to the more reactive Cu⁺ form through a single-electron transfer mechanism [27]. The reduced copper ion then exhibits high affinity for the dithiolane ring of lipoic acid moieties that are covalently attached to specific lysine residues in key metabolic enzymes [2, 57–59].

The molecular interaction between Cu⁺ and the lipoyl group occurs through coordination chemistry, where the copper ion forms stable complexes with the sulfur atoms in the dithiolane ring. This binding induces significant conformational changes in the target proteins, particularly in dihydrolipoamide S-acetyltransferase (DLAT), a core component of the pyruvate dehydrogenase complex. Structural studies reveal that copper binding disrupts the native folding of DLAT's lipoyl domains, exposing hydrophobic regions that are normally buried [60–62]. This exposure promotes interprotein interactions through hydrophobic forces and disulfide bridge formations, leading to the formation of abnormal oligomers [63, 64].

The aggregation process follows a nucleation-elongation mechanism, where initial copper-induced misfolding creates aggregation nuclei that subsequently recruit additional misfolded proteins. These aggregates exhibit amyloid-like characteristics, including β-sheet-rich structures and resistance to proteolytic degradation [65–67]. The growing aggregates physically disrupt the structural organization of the mitochondrial matrix, interfering with the efficient channeling of metabolic intermediates between enzymes in the TCA cycle.

Simultaneously, copper binding directly impacts iron-sulfur cluster proteins through multiple mechanisms. The redox-active Cu⁺ can participate in Fenton-like reactions, generating hydroxyl radicals that damage the Fe-S clusters essential for electron transport. Furthermore, copper competes with iron for binding sites in scaffold proteins during cluster assembly, resulting in the formation of unstable clusters that readily disintegrate. This disruption affects critical enzymes, including complexes I, II, and III of the electron transport chain, as well as aconitase in the TCA cycle [68, 69].

At the proteostatic level, the aggregated lipoylated proteins overwhelm the mitochondrial protein quality control system. The mitochondrial matrix chaperones, particularly mtHSP70 and HSP60, attempt to refold or disaggregate the accumulating misfolded proteins [70–72]. When these efforts fail, the mitochondrial unfolded protein response (UPRmt) is activated, involving upregulation of chaperone expression and activation of mitochondrial proteases [73–75]. However, the persistent accumulation of aggregates ultimately leads to proteostatic collapse.

The mitochondrial damage resulting from these molecular events triggers the release of mitochondrial DNA through permeability transition pore opening and outer membrane rupture. The cytosolic mtDNA containing unmethylated CpG motifs is recognized by cyclic GMP-AMP synthase (cGAS), which catalyzes the synthesis of 2′3'-cGAMP [43, 76, 77]. This second messenger activates STING on the endoplasmic reticulum membrane, initiating a signaling cascade that involves TBK1 phosphorylation and IRF3 nuclear translocation, ultimately driving type I interferon gene expression [78–80].

This detailed molecular understanding of copper-protein aggregation not only elucidates the fundamental mechanisms of cuproptosis but also provides specific targets for therapeutic intervention. The precise coordination chemistry between copper and lipoic acid, the structural determinants of protein aggregation, and the specific components of the resulting stress responses offer multiple entry points for developing targeted therapies that could modulate this cell death pathway for cancer treatment.

### Downstream signaling and execution of cuproptosis

Cuproptosis is a recently defined form of regulated cell death triggered by intracellular copper overload. Its downstream signaling pathways and execution mechanisms are complex and multi-layered, involving mitochondrial dysfunction, proteostatic disruption, immune modulation, and metabolic reprogramming. Understanding these mechanisms is crucial for developing therapeutic strategies targeting cuproptosis. The execution of cuproptosis can be broadly categorized into mitochondria-dependent and mitochondria-independent mechanisms, both of which lead to cellular damage and death and exert profound immunomodulatory and metabolic effects [81–83].

The core mechanism of cuproptosis is intimately tied to mitochondrial metabolism, particularly in the tricarboxylic acid (TCA) cycle and oxidative phosphorylation:

1. FDX1 functions as a critical upstream regulator by reducing Cu^2^⁺ to the more reactive Cu⁺ form. It also promotes the lipoylation of key enzymes in the TCA cycle, such as dihydrolipoamide S-acetyltransferase (DLAT), by facilitating the interaction between lipoic acid synthase (LIAS) and the glycine cleavage system H protein (GCSH). This lipoylation primes these enzymes for copper binding and subsequent aggregation [40, 84, 85].
2. Copper-Induced Protein Aggregation and Fe-S Cluster Destabilization: Cu⁺ directly binds to lipoylated residues on DLAT and other TCA enzymes, inducing abnormal oligomerization and aggregation. Concurrently, mitochondrial Cu⁺ disrupts iron-sulfur (Fe-S) cluster proteins, which are essential for electron transport and enzymatic activity in both the mitochondrial respiratory chain and multiple metabolic pathways. This dual insult leads to proteotoxic stress, metabolic collapse, and elevated reactive oxygen species (ROS) [86].
3. Activation of Heat Shock Response: the accumulation of misfolded and aggregated proteins triggers a heat shock response characterized by upregulation of HSP70 and other molecular chaperones. This transcriptional program reflects an overwhelmed protein quality control system and further propagates cuproptosis signaling [47, 48, 87–90].

In summary, mitochondria-dependent cuproptosis revolves around the FDX1–LIAS–DLAT axis, where copper-induced lipoylated protein aggregation and Fe-S cluster loss converge to cause irreversible metabolic failure.

Emerging evidence suggests that cuproptosis can also be executed through extramitochondrial pathways. (1) Cytoplasmic Proteotoxicity via UPS Inhibition: The disulfiram/copper (DSF/Cu) complex induces aggregation of the p97–NPL4 segregase adapter, a key component of the ubiquitin–proteasome system (UPS). This impairs protein degradation, leading to proteotoxic stress and cell death independent of mitochondrial respiration [91–93]. (2) FDX1-Independent Pathways: In certain cell types such as astrocytes, the histone H3–H4 tetramer can function as an alternative Cu^2^⁺ reductase, facilitating copper-induced toxicity even in the absence of FDX1, highlighting the metabolic plasticity of cuproptosis execution [94, 95].

Thus, cuproptosis exhibits organelle plasticity, with both mitochondrial and cytoplasmic pathways contributing to its lethal effects.

Cuproptosis is not merely a cell-intrinsic process; it actively engages the immune system, making it a potential target for immunotherapy. (1) mtDNA Release and cGAS–STING Activation: mitochondrial damage during cuproptosis leads to the release of mitochondrial DNA (mtDNA) into the cytosol, activating the cGAS–STING pathway. This triggers type I interferon production and enhances dendritic cell maturation, CD8⁺ T cell infiltration, and NK cell recruitment, fostering an immunogenic tumor microenvironment [43, 76, 77, 96–99]. (2) Immunogenic Cell Death (ICD): cuproptotic cells release damage-associated molecular patterns (DAMPs), including ATP, HMGB1, and calreticulin, which promote antigen presentation and sustained antitumor immunity [100–107]. (3) Counter-Regulatory PD-L1 Upregulation: paradoxically, cuproptosis can also induce PD-L1 expression via JAK/STAT signaling or PARP1/GSK-3β-mediated stabilization, suggesting an adaptive immune resistance mechanism [43, 47, 108–111]. This supports the rationale for combining cuproptosis inducers with immune checkpoint inhibitors.

Sensitivity to cuproptosis is modulated by metabolic and epigenetic factors:

1. p53-Mediated Metabolic Rewiring: p53 suppresses glycolysis and enhances oxidative phosphorylation, increasing cellular reliance on mitochondrial respiration and thereby sensitizing cells to cuproptosis [112–114]. p53 also inhibits NADPH production, reducing glutathione levels and amplifying copper-induced oxidative stress [115–120].
2. METTL16 Lactylation and m⁶A Modification: Copper promotes lactylation of METTL16 at lysine 229, enhancing its methyltransferase activity and facilitating m⁶A modification of FDX1 mRNA [65, 83]. This post-transcriptional upregulation of FDX1 creates a positive feedback loop that reinforces cuproptosis susceptibility.

Cuproptosis represents a unique form of regulated cell death driven by copper-dependent disruption of metabolic enzymes and proteostasis, with significant immunostimulatory potential. Its dual role in activating antitumor immunity while occasionally eliciting immune checkpoint upregulation underscores its therapeutic complexity. A deeper understanding of these downstream signal transduction and execution mechanisms will facilitate the translation of cuproptosis-targeting strategies into clinically effective anticancer therapies, particularly in combination with metabolic modulators and immunotherapy.

## Interplay between copper homeostasis and cuproptosis

### Disruption of homeostasis leading to cuproptosis

Copper is an indispensable trace element that functions as a catalytic cofactor for a plethora of enzymes, known as cuproenzymes, which are vital for fundamental cellular processes, including mitochondrial respiration (e.g., cytochrome c oxidase) [29, 32, 121, 122], antioxidant defense (e.g., superoxide dismutase, SOD1), and neurotransmitter synthesis. However, the very property that makes copper an excellent biocatalyst—its ability to cycle between oxidized (Cu^2^⁺) and reduced (Cu⁺) states—also renders it potentially cytotoxic. In its free ionic form, copper can catalyze the generation of reactive oxygen species (ROS) via Fenton-like reactions and cause indiscriminate damage to proteins, lipids, and nucleic acids [49, 123, 124]. To harness the benefits of copper while mitigating its risks, cells have evolved a sophisticated and highly regulated network of proteins to maintain copper homeostasis. The recent discovery of cuproptosis, a novel form of regulated cell death triggered by excessive copper, has profoundly illuminated the critical nature of this homeostatic balance.

Cellular copper homeostasis is maintained through four tightly coordinated processes: uptake, chaperoning, efflux, and storage. This system ensures that the intracellular "labile copper pool"—the biologically active, chelatable fraction—is kept at an extremely low, non-toxic concentration. Uptake: Copper entry into the cell is primarily mediated by the high-affinity copper transporter 1 (CTR1; encoded by *SLC31A1*) on the plasma membrane [15, 16, 125–127], which facilitates the uptake of Cu⁺. Recent groundbreaking research has identified the zinc transporter protein ZnT1 (*SLC30A1*) as a novel importer for Cu^2^⁺ [128–130], revealing an unexpected layer of complexity in copper acquisition and a potential point of crosstalk with zinc metabolism. Intracellular Trafficking and Chaperoning: Upon entry, free copper is virtually undetectable in the cytosol due to its immediate binding to a set of specific metallochaperones. These proteins function as dedicated delivery trucks, shuttling copper to distinct cellular compartments and target proteins without releasing it into the cytosol. ATOX1 delivers copper to the copper-transporting P-type ATPases including ATP7A and ATP7B [17, 131, 132], located in the trans-Golgi network (TGN). These ATPases load copper into copper-enzymes within the secretory pathway. COX17 is specialized for transporting copper to the mitochondria, where it is incorporated into cytochrome c oxidase (COX), a critical complex of the electron transport chain [29, 32, 121, 122]. CCS (Copper Chaperone for SOD1) directly delivers copper to superoxide dismutase 1 (SOD1) [28, 133–136], enabling its maturation and antioxidant activity. Efflux and Storage: To prevent accumulation, cells possess robust mechanisms for copper sequestration and export. ATP7A and ATP7B are the primary efflux pumps [23, 137–141]. Under conditions of copper excess, they traffic from the TGN to vesicular compartments or the plasma membrane to facilitate copper excretion from the cell [23, 131]. ATP7B is particularly crucial in hepatocytes for biliary copper excretion. Metallothioneins (MTs) and glutathione (GSH) act as high-capacity molecular buffers [21, 142–146]. These cysteine-rich molecules chelate excess copper ions, effectively detoxifying them and maintaining the labile copper pool at a minimal level. This buffering capacity is the cell's first line of defense against transient copper fluctuations.

In many cancer types, this precise homeostatic balance is disrupted, leading to a state of relative copper overload, a phenomenon sometimes referred to as "cuproplasia." Oncogenic signaling can upregulate the expression of copper importers like SLC31A1 or impair the function [147–149] or trafficking of efflux pumps like ATP7B [150]. This acquired dependence on copper is driven by the metal's role as a cofactor for pro-tumorigenic enzymes involved in angiogenesis (e.g., LOX), cell proliferation, and metastasis [105, 151–154].

The transition from cuproplasia to cuproptosis occurs when the homeostatic system is overwhelmed. Once the buffering capacity of metallothioneins (MTs) and glutathione (GSH) is exhausted, and efflux mechanisms prove insufficient, the labile copper pool expands dramatically. This surge in bioavailable copper, particularly within mitochondria, sets the stage for cuproptosis. Therefore, copper homeostasis acts as a gatekeeper; its failure is the essential permissive condition for the activation of the cuproptosis pathway.

**Fig. 3:**
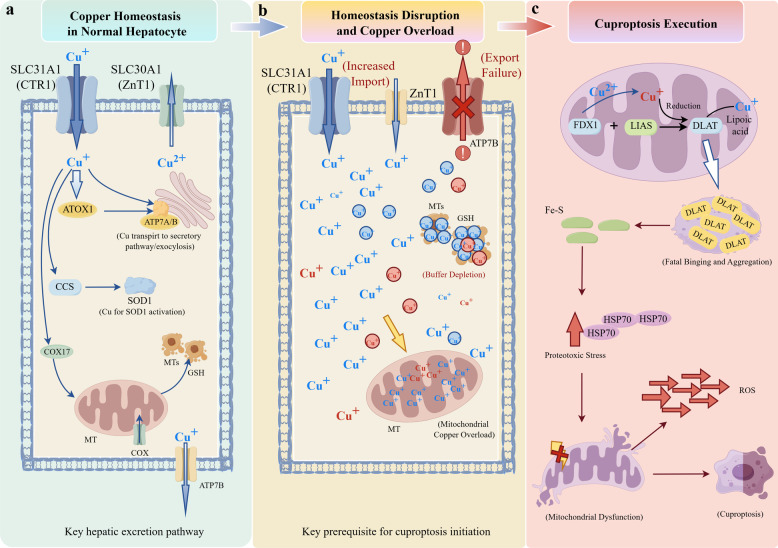
How Disruption of Homeostasis Leads to Cuproptosis (by Figdraw) This illustration delineates the copper (Cu) homeostasis mechanism in normal hepatocytes (**a**), and how its dysregulation under pathological conditions such as cancer leads to mitochondrial Cu overload (**b**). This is followed by FDX1-mediated incorporation of Cu^+^ into the lipoylation sites of DLAT, triggering protein aggregation, disruption of Fe-S clusters, and proteotoxic stress, ultimately driving the sequential progression toward cuproptosis (**c**)

In summary, the relationship between copper homeostasis and cuproptosis is one of profound interdependence and tension. The homeostatic apparatus—comprising importers, chaperones, buffers, and exporters—functions as a sophisticated system to permit the essential utilization of copper while constraining its lethal potential. The dysregulation of this very system in cancer creates the permissive environment of copper overload. The execution of cuproptosis is then not a random toxic event but a direct consequence of this dysregulation, channeled through the specific, FDX1-mediated disruption of mitochondrial metabolism. This refined understanding reveals the nodes of copper homeostasis (Fig. 3).

### Metabolic dependencies

Cuproptosis, a recently elucidated form of copper-dependent regulated cell death, highlights the intricate link between cellular copper homeostasis and mitochondrial metabolism. Unlike other metal-driven death pathways, cuproptosis is characterized by the direct binding of copper to lipoylated enzymes in the tricarboxylic acid (TCA) cycle, particularly dihydrolipoamide transacetylase (DLAT), leading to protein aggregation, loss of iron–sulfur (Fe–S) cluster proteins, and proteotoxic stress. However, the efficacy of cuproptosis is often compromised by the adaptive metabolic landscape of the tumor microenvironment (TME), including hypoxia and elevated glutathione (GSH) levels [155, 156], which collectively promote resistance to copper-mediated cytotoxicity [9, 43, 127].

Central to this metabolic interplay is the mitochondrial oxidative phosphorylation (OXPHOS) system [157], which not only drives ATP production but also relies heavily on Fe–S cluster proteins for electron transport across complexes I–III [158, 159]. In many cancers, upregulated OXPHOS supports tumor growth and survival, yet it also renders cells vulnerable to metabolic disruption [160–163]. Recent advances suggest that targeting both copper delivery and mitochondrial respiration can synergistically enhance cuproptosis.

Qin et al. [164, 165] identified that a copper–shikonin coordination network coupled with the OXPHOS inhibitor atovaquone can orchestrate a “dual metabolic interference” strategy. This approach simultaneously induces copper overload and suppresses complex III activity, thereby reducing oxygen consumption and exacerbating the metabolic crisis. In particular, the interplay extends beyond mere copper accumulation. The release of Cu⁺ ions in the TME catalyzes Fenton-like reactions, generating hydroxyl radicals (•OH) that synergize with shikonin-induced oxidative stress to promote mitochondrial membrane depolarization and caspase-3-dependent apoptosis. Furthermore, the depletion of GSH—a key copper chelator—by the nanoplatform enhances copper bioavailability and potentiates DLAT aggregation, thereby reinforcing cuproptosis.

These findings underscore that cuproptosis is not an isolated process but is deeply embedded in the metabolic network of the cell. The dependency on Fe–S cluster integrity and mitochondrial respiration reveals new therapeutic vulnerabilities. By targeting copper homeostasis and OXPHOS, it is possible to overcome TME-driven resistance and achieve potent antitumor effects, as evidenced by significant tumor suppression in vivo.

Recent studies have begun to unravel how cancer cells rewire their metabolic programs to evade cuproptosis, highlighting a critical dependency on specific metabolic pathways that intersect with copper handling. In colorectal cancer (CRC), the cyclin-dependent kinase inhibitor 2 A (CDKN2A) has emerged as a key regulator linking copper homeostasis, metabolic reprogramming, and cuproptosis resistance. Single-cell and spatial transcriptomic analyses reveal that CDKN2A is markedly upregulated during tumor progression [166–169], coinciding with an enhanced capacity to resist copper-induced cell death. This upregulation is driven not only by transcriptional activation via MEF2D but also through post-transcriptional mechanisms involving the SNHG7/miR-133b axis, underscoring a multi-layered regulatory network that fine-tunes CDKN2A expression [170].

Functionally, CDKN2A orchestrates a metabolic shift toward glycolysis, thereby bypassing copper-dependent mitochondrial toxicity [171]. CDKN2A-positive tumor epithelial cells exhibit elevated activity in glycolytic pathways, including the fructose-2,6-bisphosphate-mediated activation of phosphofructokinase-1 (PFK-1), a rate-limiting enzyme in glycolysis [172, 173]. Knockdown of CDKN2A results in reduced expression of PFK isoforms (PFKL and PFKM), impairing glycolytic flux and sensitizing cells to cuproptosis [174, 175]. This metabolic diversion illustrates how cancer cells exploit energy metabolism to mitigate copper-induced stress [176–179], a strategy distinct from other metal-dependent cell death pathways. Concurrently, CDKN2A modulates copper ion dynamics by regulating the expression of copper transporters. Silencing CDKN2A leads to upregulated expression of the copper importer SLC31A2 and downregulation of the exporter ATP7B [180–182], resulting in intracellular copper accumulation and heightened cuproptosis sensitivity. This dual role of CDKN2A in both glycolytic activation and copper efflux underscores its central position at the nexus of metabolic and ion homeostasis.

Moreover, CDKN2A-driven cuproptosis resistance is coupled with pro-tumorigenic phenotypes, including epithelial-mesenchymal transition (EMT) and Wnt pathway activation [170, 183]. These processes are further supported by a unique tumor microenvironment enriched with SPP1 + tumor-associated macrophages and MMP7, facilitating immune evasion and matrix remodeling [170, 184].

In summary, the interplay between copper homeostasis and cuproptosis is profoundly influenced by metabolic dependencies, particularly glycolysis, which provides a protective mechanism against copper toxicity. CDKN2A serves as a pivotal mediator of this crosstalk, integrating transcriptional, metabolic, and ion-regulatory signals to promote tumor survival and progression. Targeting CDKN2A or its downstream effectors may therefore offer novel therapeutic opportunities to reactivate cuproptosis in resistant cancers, especially when combined with agents that disrupt copper homeostasis.

Cuproptosis is characterized by mitochondrial copper overload, which disrupts the tricarboxylic acid (TCA) cycle through abnormal oligomerization of dihydrolipoamide S-acetyltransferase (DLAT), a key component of the pyruvate dehydrogenase complex [5, 9, 185]. This oligomerization is facilitated by the reduction of Cu^2^⁺ to Cu⁺ by ferredoxin 1 (FDX1), leading to proteotoxic stress and ultimately cell death [186, 187]. Notably, the TCA cycle serves as a metabolic hub that integrates multiple nutrient inputs, including those from branched-chain amino acid (BCAA) catabolism [188].

In pancreatic ductal adenocarcinoma (PDAC), BCAA metabolism is frequently reprogrammed to support rapid tumor growth and survival under nutrient stress. BCAA catabolism—catalyzed by branched-chain amino acid transaminase 1 (BCAT1) and the branched-chain α-keto acid dehydrogenase complex (BCKDHC)—feeds intermediates into the TCA cycle, sustaining energy production and biosynthetic precursors [189–191]. Inhibition of BCAT1 by agents such as gabapentin not only disrupts BCAA metabolism but also enhances reactive oxygen species (ROS) production, further sensitizing cancer cells to metabolic stress [192–198].

The convergence of copper toxicity and BCAA metabolic inhibition represents a synergistic antitumor strategy. As demonstrated by Shi et al., a copper-doped mesoporous silica nanoplatform (XQ/Gp@CMSNs) co-delivering gabapentin and Cu^2^⁺ effectively targets PDAC cells via the transferrin receptor (CD71) [199–202]. Within the acidic tumor microenvironment, the nanoparticles release Cu^2^⁺ and gabapentin, leading to mitochondrial copper accumulation and BCAT1 suppression. This dual action induces DLAT oligomerization, FDX1 downregulation, and TCA cycle disruption, thereby activating cuproptosis while simultaneously impairing BCAA-driven energy metabolism [203].

Metabolomic analyses of treated tumors confirmed significant alterations in central carbon metabolism and BCAA-related pathways, underscoring the metabolic synergy between copper-induced toxicity and BCAA catabolism inhibition [203]. Importantly, this approach minimizes systemic toxicity by leveraging tumor-specific targeting and microenvironment-responsive drug release.

Cuproptosis is triggered by the accumulation of intracellular copper, which binds directly to lipoylated enzymes in the tricarboxylic acid (TCA) cycle, leading to their aggregation, loss of iron-sulfur cluster proteins, and proteotoxic stress [9]. Key among these enzymes is dihydrolipoamide S-acetyltransferase (DLAT), a component of the pyruvate dehydrogenase complex [204]. The aggregation of lipoylated proteins disrupts mitochondrial integrity and function, ultimately inducing cell death. Notably, cells with high dependency on mitochondrial respiration are more susceptible to cuproptosis. This metabolic preference was demonstrated by Tsvetkov et al. [9], who observed that cells relying predominantly on oxidative phosphorylation showed heightened sensitivity to copper ionophores compared to those utilizing glycolysis. This suggests that cuproptosis is not merely a passive consequence of copper overload but an active process contingent upon the metabolic wiring of the cell.

The reductase FDX1 serves as an upstream regulator of protein lipoylation and is essential for cuproptosis. It facilitates the reduction of Cu^2^⁺ to Cu⁺, which subsequently binds to lipoylated TCA cycle proteins [9, 205]. Elevated FDX1 expression correlates with increased protein lipoylation and enhanced copper sensitivity [206]. Moreover, FDX1 has been implicated in the regulation of mitochondrial function and oxidative stress responses, further bridging copper metabolism with cellular energy pathways.

Additionally, copper chaperones such as COX17 and antioxidant 1 copper chaperone (ATOX1) coordinate the delivery of copper to mitochondrial enzymes like cytochrome c oxidase (COX) and superoxide dismutase (SOD1) [207–211], both critical for energy production and redox balance. Dysregulation of these chaperones not only impairs metabolic enzyme activity but also modulates cellular susceptibility to copper-induced death.

The interplay between copper homeostasis and cuproptosis opens new avenues for targeting metabolically active cells, particularly in cancers characterized by high mitochondrial respiration. For instance, drugs such as elesclomol—a copper ionophore—selectively induce cuproptosis in tumor cells with elevated oxidative phosphorylation [47]. Similarly, compounds like 4-octyl itaconate have been shown to inhibit glycolysis and promote cuproptosis by targeting glyceraldehyde-3-phosphate dehydrogenase (GAPDH) [212, 213], thereby shifting cellular metabolism toward a cuproptosis-sensitive state. Furthermore, the expression of cuproptosis-related genes (CRGs) such as DLAT, FDX1, and SLC31A1 has been linked to tumor metabolism, immune infiltration, and patient prognosis across multiple cancer types [148, 205, 214, 215]. These genes not only serve as prognostic biomarkers but also represent potential therapeutic targets for modulating copper-induced cell death in a metabolic context.

## Copper dysregulation and cuproptosis in diseases

Copper dyshomeostasis and the recently delineated form of regulated cell death, cuproptosis, are increasingly recognized as contributing factors in the pathogenesis of a broad spectrum of human disorders. While the liver serves as the central hub for copper metabolism and a primary site for related pathophysiology, the implications of this metallostatic axis extend far beyond hepatic conditions. The subsequent sections will systematically examine the roles of copper homeostasis and cuproptosis in neurodegenerative diseases, cardiovascular disorders, and diabetic kidney disease. This will be followed by a focused and detailed analysis of their distinct mechanisms within the context of various liver diseases, ranging from metabolic dysfunction-associated steatotic liver disease to hepatocellular carcinoma.

### Neurodegeneration

Copper (Cu) is a vital redox-active metal whose homeostasis is crucial for neuronal function. Manifesting as regional deficiency or excess, dysregulation of cerebral copper balance is a common feature across major neurodegenerative diseases. The pathogenic mechanisms, while varying in their initial triggers, converge on shared downstream cascades of proteotoxicity, bioenergetic failure, and regulated cell death.

In AD, Cu accumulates within amyloid plaques and promotes both amyloid-β (Aβ) and tau pathology through distinct mechanisms. Cu^2^⁺ coordinates with Aβ, particularly the Aβ42 species, to stabilize neurotoxic oligomers and fibrils. This Cu-Aβ complex exhibits enhanced redox cycling, generating hydroxyl radicals via Fenton chemistry that exacerbate oxidative damage [216, 217]. Concurrently, Cu facilitates the hyperphosphorylation of tau, promoting its aggregation into neurofibrillary tangles. Crucially, elevated intracellular Cu can directly engage the cuproptosis pathway by binding to lipoylated enzymes in the mitochondrial tricarboxylic acid (TCA) cycle, leading to toxic protein aggregation, loss of iron-sulfur clusters, and neuronal death. Strategies using Cu chelators (e.g., PBT2) mitigate pathology in models by sequestering labile Cu and disrupting these metal-centered toxic processes [218–220].

In PD, Cu potently accelerates the pathological aggregation of α-synuclein (α-Syn) [220]. Binding of Cu^2^⁺ to the N-terminal domain reduces electrostatic repulsion between α-Syn monomers, dramatically increasing the rate of fibrillation. The resulting Cu-α-Syn complexes possess pro-oxidant activity, catalyzing the oxidation of dopamine to cytotoxic quinones. Within vulnerable dopaminergic neurons of the substantia nigra, Cu overload impairs mitochondrial respiration and sensitizes cells to cuproptosis, a process likely amplified by the characteristic depletion of the intracellular Cu buffer glutathione (GSH) [221, 222]. Genetic variants in Cu transporters (ATP7A, SLC31A1) further link systemic Cu handling to PD susceptibility [223].

Familial ALS linked to SOD1 mutations provides a direct connection between Cu metabolism and neurodegeneration [224]. Many mutations destabilize the SOD1 apo-protein, impairing proper binding of its catalytic Cu and Zn ions. This metal-deficient state promotes SOD1 misfolding and aggregation [225]. The mutant protein can also aberrantly interact with the Cu chaperone CCS and disrupt normal cellular Cu distribution, creating a state of secondary Cu dyshomeostasis [226]. The consequent mitochondrial dysfunction, combined with an increased Cu burden, primes motor neurons for cuproptosis. Therapeutic approaches that restore Cu delivery to SOD1 (e.g., Cu (II) (atsm)) or chelate excess Cu show efficacy in preclinical models, validating Cu homeostasis as a therapeutic target [227].

In HD, Cu accumulates in the striatum and directly interacts with the N-terminal domain of mutant huntingtin (mHTT), promoting its oligomerization [228]. Furthermore, Cu potently inhibits key metabolic enzymes like lactate dehydrogenase (LDH), disrupting the astrocyte-neuron lactate shuttle and exacerbating bioenergetic deficits in striatal neurons [229, 230]. This combination of proteotoxic stress (mHTT aggregation) and metabolic compromise creates a cellular environment where subsequent Cu overload can efficiently activate the cuproptosis execution pathway, driving the selective degeneration characteristic of HD [231].

The interplay between Cu dyshomeostasis and neurodegenerative pathogenesis is multifaceted, involving direct metal-protein interactions, redox chemistry, and the activation of a dedicated mitochondrial cell-death pathway. Cuproptosis emerges as a critical mechanistic link, translating the biochemical insult of Cu overload into irreversible neuronal loss. This consolidated view highlights the central role of metallostasis in brain health and positions the Cu homeostasis network as a compelling target for developing disease-modifying therapies across the spectrum of neurodegeneration.

### Cardiovascular diseases

The pathogenesis of cardiovascular diseases (CVD) is profoundly influenced by underlying metabolic disorders such as diabetes mellitus and obesity. Emerging evidence positions the disruption of copper homeostasis and the subsequent activation of related pathways [2] as a critical molecular nexus linking metabolic dysfunction to cardiac and vascular injury, extending beyond traditional risk factors.

A primary mechanistic link involves mitochondrial dysfunction and an energetic crisis. Metabolic syndrome and hyperglycemia induce systemic oxidative stress and alter nutrient-sensing pathways, which can impair the biosynthesis and metalation of mitochondrial cuproenzymes, particularly cytochrome c oxidase (CCO) [232]. This results in inefficient oxidative phosphorylation, a state of energy depletion that exacerbates cardiac contractile dysfunction and predisposes cardiomyocytes to various insults. Concurrently, the metabolic milieu of insulin resistance and hyperlipidemia promotes a state of chronic, low-grade inflammation. Copper acts as a potent modulator of inflammatory signaling; it can activate the NLRP3 inflammasome in macrophages and vascular cells, leading to the maturation and secretion of IL-1β and IL-18 [52, 233]. This perpetuates endothelial dysfunction, promotes atherosclerotic plaque instability, and contributes to myocardial remodeling.

Crucially, the metabolic dysregulation itself can directly disrupt copper homeostasis. In diabetic states, for instance, hyperglycemia and the accumulation of advanced glycation end products (AGEs) have been shown to upregulate the expression of the high-affinity copper transporter SLC31A1 (CTR1), leading to aberrant intracellular copper accumulation in cardiovascular cells [48]. This copper overload creates a feed-forward loop of oxidative stress via Fenton reactions and provides the substrate for copper-dependent signaling dysregulation.

The intersection of these pathways—mitochondrial stress, inflammation, and copper dyshomeostasis—converges to create a cellular environment highly susceptible to specific forms of damage. It is within this context that the process of cuproptosis gains pathophysiological relevance in CVD. While the detailed mechanism of cuproptosis involves lipoylated protein aggregation in the TCA cycle, its initiation is fundamentally dependent on the excessive intracellular bioavailable copper that results from the metabolic disturbances described above. Thus, metabolic diseases establish the preconditions (copper overload, mitochondrial stress) that prime cardiovascular cells for this unique death pathway, contributing to the loss of cardiomyocytes in diabetic cardiomyopathy or endothelial cells in accelerated atherosclerosis.

In summary, metabolic diseases promote cardiovascular pathology not only through hemodynamic and lipid abnormalities but also by fundamentally altering the cellular ionomic landscape. The dysregulation of copper trafficking and redox chemistry serves as a key molecular mechanism, integrating metabolic signals with processes of inflammation, bioenergetic failure, and ultimately, cell fate decisions in the heart and vasculature. Targeting this copper-centric axis may offer a novel therapeutic strategy for mitigating cardiovascular complications in patients with metabolic syndromes.

### Diabetic kidney disease

The susceptibility of renal tubular epithelium to cuproptosis is fundamentally heightened within the metabolic dysregulation characteristic of diseases like Diabetic Kidney Disease (DKD). This predisposition arises from a multi-layered mechanistic convergence where systemic metabolic perturbations reconfigure the intracellular milieu to favor copper-mediated cytotoxicity. Core to this process is the metabolic stress-induced dysregulation of copper homeostasis, primarily via the transcriptional upregulation of the high-affinity copper importer SLC31A1 (CTR1) driven by hyperglycemia and advanced glycation end products [25]. Concurrently, the mitochondrial metabolic remodeling in nutrient-excess states—marked by altered fuel utilization and increased reliance on lipoylated TCA cycle dehydrogenases—renders the mitochondrial proteome uniquely vulnerable to copper-induced aggregation and functional sabotage. A critical amplifying mechanism involves the destabilization of iron-sulfur (Fe-S) cluster biogenesis, a process compromised by both metabolic oxidative stress and the frequent concomitant iron overload observed in metabolic syndromes [204]. This impairment directly targets the [4Fe-4S]-dependent enzyme lipoic acid synthase (LIAS), leading to deficient protein lipoylation. The resulting pool of aberrantly modified or non-lipoylated proteins not only disrupts core metabolism but also primes the cell for proteotoxic crisis upon copper binding [40, 234, 235]. Thus, the metabolic disease environment orchestrates a pathogenic triad of increased copper bioavailability, enhanced target susceptibility, and compromised protective Fe-S cluster assembly, collectively lowering the threshold for cuproptosis execution and driving progressive tubular injury.

In summary, in kidney diseases with metabolic origins, the cellular milieu is reconfigured to favor cuproptosis. Metabolic insults dysregulate copper import, remodel mitochondrial metabolism to increase target vulnerability, and disrupt Fe-S cluster biogenesis—often exacerbated by concurrent iron dyshomeostasis. This positions coppertosis not as an isolated event but as a convergent mechanism of tubular injury downstream of metabolic derangements. Therapeutic strategies aimed at re-establishing metal ion homeostasis or supporting mitochondrial Fe-S cluster integrity may therefore be particularly relevant for mitigating metabolic disease-associated kidney injury.

### Liver diseases

The delicate balance of copper homeostasis is maintained through a series of coordinated processes, including absorption, transport, storage, and excretion. Disruption in these processes might precipitate the development of liver disease [236]. Therefore, maintaining proper copper homeostasis is essential for cellular health [237, 238]. If the copper intake is deficient in the diet, lipids will be accumulated, even leading to the onset of MASLD potentially. Multiple studies with 5218 cases have demonstrated an inverse correlation between serum copper levels and the likelihood of MASLD [239]. On the contrary, when copper is excessive, the surplus Cu^+^ is typically managed by cytoplasmic metallothioneins (MTs) within hepatocytes or confined within lysosomes.

The protein ATP7B is crucial for transporting excess copper into the bile, thus preventing its accumulation in tissues [1]. MTs and glutathione (GSH) are key molecules that typically sequester excess intracellular Cu^+^.

Elevated copper ion levels can trigger apoptosis. However, excessive copper will lead to oxidative damage or improper binding to biomolecules [240]. Free copper ions usually react with oxygen to produce reactive oxygen species (ROS) [241]. Then, ROS will be accumulated through various pathways, such as affecting the synthesis of superoxide dismutase (SOD) [242] and the activity of cytochrome c oxidase (CcO) [239], which may cause oxidative stress, lipid peroxidation, and protein misfolding, ultimately leading to cellular inflammation or death. Furthermore, high concentration of Cu^+^ can disrupt the function of the transcription factor like Macrophage-1 Antigen (Mac-1), causing DNA damage, base oxidation, and inhibiting the transcription and expression of transporter protein genes, thereby initiating programmed cell death. Some studies have suggested that increased Cu^+^ levels could alter Mac-1 activity, leading to DNA damage and base oxidation, and consequently inducing programmed cell death, which is recognized as a pathway for copper-induced cell death.

#### Metabolic dysfunction-associated steatotic liver disease

From simple steatosis to non-alcoholic steatohepatitis (NASH), if left untreated, MASLD can progress to cirrhosis and hepatocellular carcinoma (HCC). Epidemiological data suggest that MASLD affects about 30% of the global population, making it the most common chronic liver disorder worldwide [243]. Recent research has recognized MASLD as a hepatic manifestation of metabolic syndrome. Patients with MASLD often exhibit reduced hepatic copper levels, which are associated with the development of hepatic steatosis. The molecular mechanisms by which copper regulates lipid metabolism, a key factor in metabolic disorders like MASLD, are not yet fully understood. It has been observed that copper repair following hepatic ceruloplasmin (CP) ablation enhances lipolysis by promoting the assembly of copper-loaded SCO1-LKB1-AMPK complexes. Overnutrition-induced elevation of CP leads to hepatic copper depletion, whereas CP ablation restores copper levels without causing hepatotoxicity and improves MASLD in mice [244]. Copper deficiency may also impair the antioxidant defense system, increasing oxidative stress and contributing to MASLD [1, 245].

However, some studies have reported a negative correlation between hepatic copper and MASLD in patients without metabolic syndrome, and findings on copper levels in MASLD patients vary [246, 247]. One study found that high blood copper concentrations were protective against MASLD in men, particularly those with mild liver disease and moderate hepatic steatosis, suggesting a possible role of sex hormones in MASLD outcomes [248].

Excessive fructose consumption has been linked to the worsening of MASLD and other metabolic disorders, possibly due to disruptions in copper homeostasis [249, 250]. Fructose is known to inhibit copper absorption by suppressing the expression of duodenal CTR1. Mice fed a high-fructose diet exhibit decreased copper levels in serum and liver [7.8 ± 1.2 μg/g from 12.3 ± 1.5 μg/g in the normal group (*P* < 0.01)] [244, 249]. Copper deficiency also inhibits Carnitine Palmitoyl Transferase-I (CPT-I), the rate-limiting enzyme in fatty acid β-oxidation, while up-regulating fatty acid synthase (FAS), a key enzyme in fatty acid synthesis, leading to increased free fatty acids and triglycerides in liver cells and subsequent steatosis [250].

Furthermore, copper deficiency is associated with oxidative stress, hepatic inflammation, and insulin resistance, all of which are significant in MASLD development [250, 251]. Copper deficiency reduces hepatic iron export by decreasing ceruloplasmin (CP) activity and the expression of hepatic iron transport proteins, leading to increased hepatic iron content and exacerbating MASLD progression through oxidative stress, insulin resistance, and hepatic inflammation [239].

Oxidative stress plays a significant role in MASLD development, and antioxidants are emerging as a promising treatment option. Recent studies have shown that natural compounds with antioxidant properties can reduce hepatic steatosis and oxidative stress and also have chelating effects on copper [252]. These findings highlight the complex relationship between copper and MASLD, indicating the need for further research to clarify copper's role in MASLD pathogenesis.

#### Alcohol-associated liver disease

Copper metabolism abnormalities play a crucial role in the progression of alcohol liver diseases (ALD) to cirrhosis. Firstly, the liver, as the central organ for copper metabolism, may experience an imbalance in copper homeostasis, potentially leading to excessive copper accumulation. This can trigger mitochondrial dysfunction, oxidative stress, and proteotoxic stress, ultimately inducing cuproptosis [253]. The key pathogenic factors in ALD are acetaldehyde and reactive oxygen species (ROS) generated during alcohol metabolism. Copper ions can catalyze ROS production through the Fenton reaction, leading to mitochondrial dysfunction and lipid peroxidation [49, 52]. This suggests that abnormal copper metabolism in the liver of ALD patients may exacerbate oxidative stress and consequently aggravate hepatocyte damage via the ferroptosis pathway. Secondly, copper is primarily excreted from the liver through bile, and alcohol consumption may interfere with bile secretion. For instance, patients with alcoholic cirrhosis often exhibit abnormal expression of ceruloplasmin (CP), which may be associated with impaired function of the copper transporter ATP7B, mimicking the pathological mechanism observed in Wilson's disease (WD) [254]. Recent studies have shown that cuproptosis can regulate hepatic stellate cell (HSC) activation and then participate in liver fibrosis. For instance, the active ingredient from garlic, diallyl trisulfide (DATs), induces copper-mediated death in hepatic stellate cells (HSCs), suppresses lipid droplet autophagy, and decelerates the fibrotic process [255]. Given that the progression of ALD to liver fibrosis is closely associated with HSC activation, copper-mediated death may emerge as a potential target for intervening in alcoholic liver fibrosis [256]. Abnormal expression of copper transporters (ATP7B), chaperones (ATOX1), and regulatory factors such as FDX1 is linked to various liver diseases. For instance, the knockout of FDX1 can suppress cuproptosis; however, whether similar changes in gene expression occur in patients with ALD remain to be further investigated. Future preclinical studies are needed to elucidate the specific role of cuproptosis in ALD and its potential for intervention.

#### Inherited metabolic hepatitis

WD is a recessive genetic disorder caused by mutations in the ATP7B gene, which encodes a copper-transporting ATPase [257, 258]. This condition is characterized by protein abnormalities affecting the liver, nervous system, and kidneys.

**Fig. 4:**
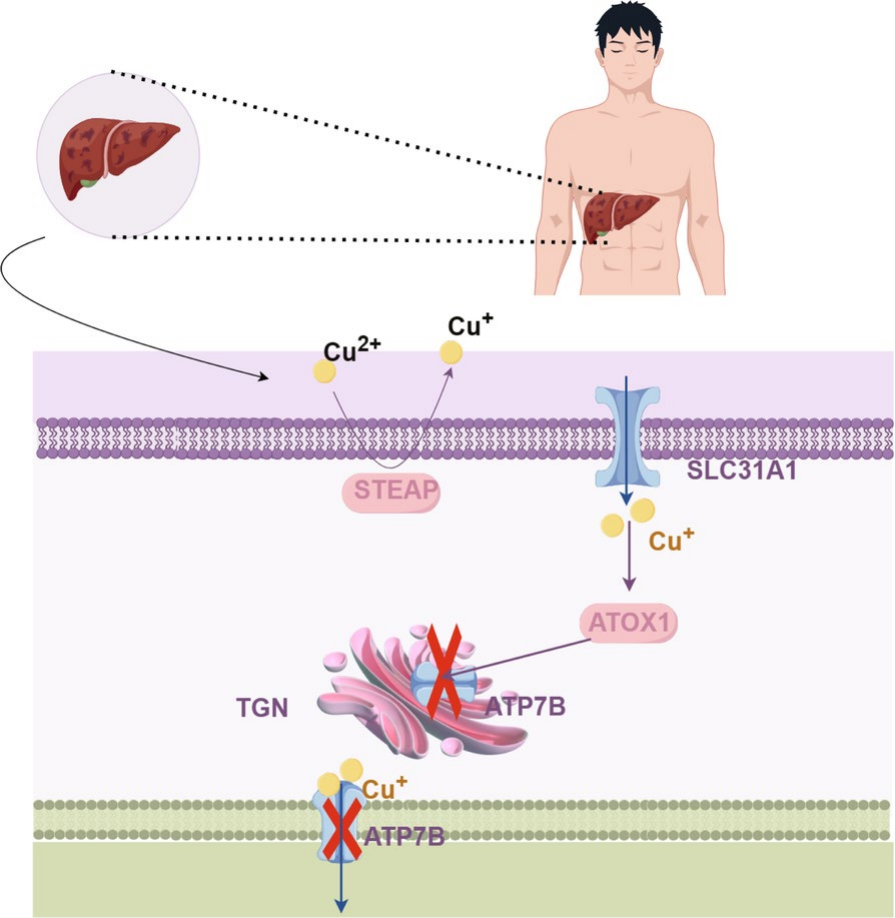
Liver fibrosis/cirrhosis caused by a genetic mutation in the ATP7B gene (by Figdraw) Copper ions are internalized by the cell through the SLC31A1 transporter protein, where they subsequently bind to ATOX1. Following this interaction, the copper ions are transported to the TGN via the ATP7B protein. Inactivation of ATP7B disrupts the excretion of copper into bile, resulting in copper accumulation within the body, which may ultimately cause hepatotoxicity

Oxidative stress, primarily driven by reactive oxygen species (ROS), is a major cause of liver injury in WD [259]. Disruptions in copper metabolism can initiate liver fibrosis, a critical precursor to cirrhosis [260]. The inactivation of ATP7B impairs biliary copper excretion, leading to copper accumulation and hepatotoxicity [261, 262] (Fig. 4). Consequently, patients may progress to hepatitis, liver failure, and cirrhosis.

Epigenetic factors, including dietary influences, contribute to the liver pathology associated with WD. Avoidance of a high-copper diet is a primary concern in the management of WD, as patients must prevent the accumulation of copper to avoid toxicity, as excessive copper absorption can induce mitochondrial oxidative stress and hepatocyte damage [263].

Such a diet may precipitate disease progression from fatty liver to steatohepatitis, a severe form of liver disease. WD is one of the few genetic disorders that can be treated effectively. Early-stage patients are often managed with dietary copper restrictions and oral medications, such as copper chelators and zinc, to slow disease progression. Advanced-stage patients may require more aggressive interventions, including plasma exchange and liver transplantation, to improve their life expectancy [264].

#### Viral hepatitis

Research has shown that copper acts as a coenzyme in the fight against chronic liver disease and hepatic fibrosis, particularly during severe liver injury and acute hepatitis, where it plays a role in collagen production [265]. As chronic hepatitis evolves into cirrhosis, there is a characteristic drop in blood levels of calcium, magnesium, phosphorus, and zinc, while copper levels tend to increase. These fluctuations in essential trace elements can be signal conditions such as liver malfunction, cholestasis, hepatic fibrosis, or liver regeneration. Trace elements, particularly copper (Cu) and iron (Fe), have been identified as significant in liver disease, especially in advanced liver degeneration [266]. Studies have found that individuals with hepatitis C virus (HCV) infection often have higher serum levels of copper and iron, with even more pronounced increases in cirrhotic patients [267, 268]. The progression of liver fibrosis is accompanied by a rise in intrahepatic copper levels, which may exacerbate HCV infection [269]. Elevated serum copper levels are also associated with a potential weakening of the body's defense mechanisms. In conditions like micronodular cirrhosis and hepatitis, excess copper can accumulate in the bloodstream due to impaired biliary excretion. Copper in the liver is primarily bound to metallothionein, which could lead to copper overload [270, 271]. Additionally, an increase in essential transition metals in HCV-positive patients has been linked to markers of oxidative stress. Similar findings of elevated serum transition metals have been observed in patients with hepatitis B virus (HBV) at various disease stages, with those who also have hepatocellular carcinoma showing even higher serum copper levels compared to controls [272]. A study focusing on female patients revealed elevated serum and urine levels of copper and iron in those with viral hepatitis A, B, C, D, and E. Among these, copper levels in scalp hair were most significantly associated with hepatitis viruses, particularly for types A, B, and C [273].

These findings suggest that viral liver injury can disrupt systemic copper homeostasis. The increase in serum or plasma copper might be a response to acute infection or copper cyanogenesis [274]. A study tracking the progression of chronic HCV liver disease noted that liver copper levels increased with the severity of fibrosis and were positively correlated with bilirubin. Conversely, a negative correlation was observed between copper and albumin levels [275].

#### Cholestatic hepatitis

Cholestatic liver disorders, such as primary biliary cirrhosis (PBC), primary sclerosing cholangitis (PSC), and the co-occurrence with autoimmune hepatitis, can cause significant pathological effects on the liver and biliary system [276]. These conditions often necessitate lifelong treatment, with liver transplantation being the ultimate therapeutic option for many patients. If untreated, PBC and PSC may progress to decompensated cirrhosis and liver failure. The liver and bile ducts are the primary homeostatic organs that regulate the excretion of manganese (Mn) and copper (Cu) [277]. Cholestasis, a hallmark of these disorders, can lead to copper accumulation in the body, which is particularly toxic to the basal ganglia of the central nervous system (CNS) [278]. While copper is an essential trace element required for various physiological processes, its accumulation, whether due to exogenous or endogenous factors, can result in severe tissue and organ damage (Table 1).

**Table 1:** Hepatic fibrosis and cirrhosis resulting from disruptions in copper homeostasis and the underlying mechanisms

**Copper Imbalance Classification**	**Disease classification**	**Pathogenesis**	**Pathogenic copper proteins**	**References**
Deficiency	MAFLD	Inhibition of PDE3B leads to a decrease in cAMP-dependent lipolysis and affects plasma cholesterol and lipoprotein levels. Ceruloplasmin (CP) upregulation reduces liver copper and causes MAFLD via SCO1/LKB1/AMPK.	PDE3B, CP	[18, 54]
Overload	Hepatitis (HBV, HCV)	May be caused by acute infection and Ceruloplasmin (CP) production.	CP	
	Wilson's disease (WD)	ATP7B7 mutation, copper accumulation in the liver and resulting toxicity.	ATP7B	[37, 38]
	Primary biliary cirrhosis (PBC) primary sclerosing cholangitis (PSC)	Cholestasis causes copper to accumulate in the organism.		[35, 42]
	Liver fibrosis (HSC)	Dysregulated copper homeostasis, leading to HSC-specific cuproptosis via the RAB18/CPT1A/DLD axis and mitochondrial proteotoxicity.	RAB18/CPT1A/DLD	[255]
	Hepatic cell carcinoma (HCC)	Copper acts as a direct allosteric activator of key kinases, including MEK1 and RAF1 within the MAPK/ERK pathway and PI3K in the AKT/mTOR axis. Copper-dependent enzymes and copper-induced oxidative stress can alter histone modification patterns and DNA methylation, potentially resulting in the silencing of tumor suppressor genes, including CDKN2A/p16INK4a.	MAPK/ERK, PI3K, CDKN2A/p16INK4a	[169, 279–283]

#### Liver fibrosis

The mechanistic link between copper homeostasis and liver fibrosis has emerged as a promising therapeutic frontier, centered on the selective induction of cuproptosis in hepatic stellate cells (HSC). Fibrotic livers exhibit a characteristic accumulation of copper, which can be therapeutically harnessed to trigger HSC-specific cell death [284, 285]. The core mechanism involves a profound disruption of mitochondrial proteostasis: excess copper ions directly bind to lipoylated components of the tricarboxylic acid (TCA) cycle, driving their toxic oligomerization and aggregation. This respiratory-dependent process culminates in the loss of iron-sulfur cluster proteins and an irreversible proteotoxic crisis. Furthermore, the study [255] delineates a novel regulatory axis in which diallyl trisulfide (DATs) targets the small GTPase RAB18. DATs [286] binding at the K153 site induces RAB18 liquid–liquid phase separation (LLPS), forming a biomolecular condensate that recruits carnitine palmitoyltransferase 1 A (CPT1A) to mitochondrial-associated membranes (MAMs) [287, 288]. The enhanced ER-mitochondria contact stabilizes MAMs, creating a calcium-enriched microenvironment that, in turn, amplifies RAB18 LLPS in a positive feedback loop. Consequently, phase-separated RAB18 inhibits lipophagy, promoting lipid droplet retention, while the co-recruited CPT1A catalyzes succinylation of dihydrolipoamide dehydrogenase (DLD) at lysine 320. This site-specific succinylation competes with ubiquitin-mediated degradation, stabilizing DLD and augmenting its enzymatic activity. The resultant enhancement of lipoic acid modification fuels the oligomerization of downstream proteins like DLAT, thereby converting the existing copper overload into a lethal cuproptosis signal within HSCs [289, 290]. This sophisticated pathway not only underscores copper's role as a pathogenic mediator in fibrosis but also validates the RAB18/CPT1A/DLD axis as a viable target for disrupting copper homeostasis in anti-fibrotic therapy.

#### Hepatocellular Carcinoma (HCC)

Copper is a vital trace element with redox activity, and it significantly affects the development of HCC. The liver, being the central organ for systemic copper regulation, is particularly susceptible to disruptions in copper homeostasis. In HCC, this often manifests as aberrant intracellular copper accumulation, which drives tumor initiation and progression through multiple interconnected mechanistic pathways [291–293].

Copper functions as a direct regulator of oncogenic signaling. It allosterically activates key kinases, including MEK1 and RAF1 in the MAPK/ERK pathway [279, 280] and PI3K in the AKT/mTOR axis [281], thereby promoting abnormal proliferation, survival, and metabolic reprogramming. Concurrently, copper's redox potential catalyzes the generation of reactive oxygen species (ROS) via the Fenton reaction [294]. Elevated ROS induces oxidative DNA damage and genomic instability, while also acting as secondary messengers to amplify pro-tumorigenic signaling networks.

Beyond that, copper remodels the tumor microenvironment to facilitate metastasis and angiogenesis. As an essential cofactor for lysyl oxidase (LOX) [295], copper drives the cross-linking of collagen and elastin, stiffening the extracellular matrix to enable invasive migration and prime pre-metastatic niches [5]. Furthermore, copper upregulates pro-angiogenic factors like VEGF, supporting neovascularization [296]. Emerging evidence also points to a cross-talk between copper and the epigenetic landscape. Copper-dependent enzymes and copper-induced oxidative stress can alter histone modification patterns and DNA methylation, potentially leading to the silencing of tumor suppressor genes such as CDKN2A/p16INK4a [169, 282, 283].

In conclusion, copper homeostasis is profoundly dysregulated in HCC, where it acts as a central node driving disease progression through direct signaling activation, oxidative stress, microenvironment remodeling, and epigenetic modulation. Restoring copper homeostasis represents a rational and promising therapeutic avenue for this malignant tumor.

Dysregulation of copper homeostasis is a pivotal driver of chemotherapy resistance in HCC, operating through multifaceted mechanisms that converge on evading cuproptosis. The hypoxic tumour microenvironment fosters adaptive resistance by stabilising HIF-1α, which transcriptionally upregulates the copper-sequestering protein metallothionein 2 A (MT2A) and reshapes metabolism via PDK1/3 to downregulate the cuproptosis target DLAT, thereby desensitising cells to copper toxicity [297]. Concurrently, classical multidrug resistance mediated by the overexpression of efflux pumps such as P-glycoprotein (P-gp) can be subverted by cuproptosis inducers, which bypass these drug extrusion pathways [298]. Notably, HCC subsets exhibiting specific metabolic vulnerabilities, such as ARID1A deficiency, demonstrate heightened dependence on mitochondrial respiration and the TCA cycle, rendering them synthetically lethal to copper overload [299]. This metabolic rewiring presents a therapeutic window for selectively eradicating otherwise resistant cells. Leveraging these insights, emerging approaches combining copper ionophores [300, 301] (elesclomol, disulfiram/copper complexes) with HIF-1α inhibitors, or deploying multifunctional copper-based nanoplatforms (metal–organic frameworks) that deplete glutathione and disrupt redox balance, offer promising avenues to overcome chemoresistance by reinstating lethal cuproptosis in HCC.

## Therapeutic opportunities: targeting the copper axis

The intricate link between copper homeostasis and cell fate, particularly through cuproptosis, has opened promising therapeutic avenues across a spectrum of diseases, notably in oncology and inherited copper metabolism disorders. Therapeutic strategies can be broadly categorized into those that deplete copper to suppress pro-tumorigenic signaling, those that exploit copper overload to induce lethal cuproptosis, those aimed at genetically restoring physiological copper handling, and innovative combination approaches designed to overcome resistance and maximize efficacy.

### Copper depletion strategies

Copper depletion strategies aim to reduce systemic or intracellular copper bioavailability, primarily targeting cancers that exhibit “cuproplasia”—a dependency on copper for growth, angiogenesis, and metastasis or treating genetic overload disorders like WD. This is achieved chiefly through chelation or competitive inhibition of absorption.

Classic copper chelators, such as D-penicillamine and tetrathiomolybdate (TM), form stable complexes with copper ions, reducing the labile copper pool. In WD, they promote urinary copper excretion, alleviating hepatic and neurological toxicity. In oncology, their utility extends beyond mere metal sequestration [302]. TM, for instance, not only chelates copper but also potently inhibits the copper chaperone ATOX1, disrupting copper transfer to the secretory pathway [303]. This downregulates the activity of cuproenzymes like lysyl oxidase (LOX), impairing extracellular matrix remodeling and angiogenesis, critical for tumor progression and metastasis [295]. Furthermore, TM can inhibit the MEK1/2 kinase pathway, adding a direct signaling blockade in cancers with specific mutations [113, 114]. Zinc salts, acting indirectly, induce intestinal metallothionein synthesis, which sequesters dietary copper and blocks its absorption, representing a well-tolerated long-term maintenance therapy for WD [63]. Thus, copper depletion serves a dual purpose: correcting pathological overload and disrupting oncogenic copper signaling networks.

### Cuproptosis induction therapy

In contrast to depletion, this strategy intentionally delivers excess copper into cells, particularly cancer cells, to trigger the novel regulated cell death pathway—cuproptosis. This leverages the intrinsic cytotoxicity of copper when homeostasis fails, offering a metabolic vulnerability for tumors reliant on mitochondrial respiration.

Key agents in this category are copper ionophores. Elesclomol (ES) functions as a dedicated copper shuttle, binding extracellular Cu^2^⁺ and delivering it to mitochondria [304]. Inside, the reductase FDX1 reduces Cu^2^⁺ to Cu⁺, which then directly binds to lipoylated TCA cycle enzymes (e.g., DLAT), inducing toxic aggregation, Fe-S cluster loss, and proteotoxic stress, culminating in cell death. ES shows particular efficacy against oxidative phosphorylation (OXPHOS)-high cancer subtypes [164, 165]. Similarly, the disulfiram/copper (DSF/Cu) complex exhibits potent cuproptosis-inducing capacity. Beyond copper ionophoric activity, DSF/Cu inhibits the proteasome via aggregating the p97-NPL4 segregase adapter, inducing ER stress, and generating reactive oxygen species (ROS), creating a multi-pronged cytotoxic insult [305]. Its ability to target cancer stem cells (via ALDH inhibition) [306] and reverse chemoresistance (via NF-κB pathway suppression) makes it a compelling repurposing candidate [307]. These inducers transform copper from a vital cofactor into a lethal weapon, with selectivity potentially guided by the metabolic profile of the tumor.

### Restoration of copper homeostasis

For diseases rooted in genetic defects of copper transport, the most definitive therapeutic approach is to correct the underlying functional deficiency, thereby restoring physiological copper homeostasis. This represents a move from symptomatic management to potential curative intervention.

The most advanced example is gene therapy for WD. VTX-801 is an investigational adeno-associated virus (AAV) vector carrying a functional copy of the ATP7B gene [308]. By transducing hepatocytes, it aims to restore biliary copper excretion, addressing the primary pathology. Preclinical studies demonstrate reduced hepatic copper accumulation and improved function, leading to its designation as an Orphan Drug and Fast Track status by regulatory agencies. Beyond monogenic disorders, future strategies may involve modulators of copper transporter or chaperone activity (e.g., specific inhibitors of CTR1 to starve tumors of copper, or stabilizers of mutant ATP7B) [309]. Pharmacologically fine-tuning the expression or function of specific nodes within the copper homeostasis network represents a frontier for precision medicine in both genetic and acquired diseases characterized by copper dysregulation.

### Novel therapeutic strategies and combination therapies

The complexity of the disease microenvironment and adaptive resistance mechanisms often limits the efficacy of monotherapies. The most promising contemporary research focuses on rational combination strategies and sophisticated delivery platforms to enhance cuproptosis, overcome resistance, and stimulate antitumor immunity.

Combination with Metabolic Modulators: To force cancer cells into a copper-susceptible state, copper ionophores are combined with agents that shift metabolic dependencies. This includes OXPHOS inhibitors [310] (e.g., atovaquone) to increase mitochondrial copper retention and stress, or glycolysis inhibitors [213, 311] (e.g., 4-octyl itaconate targeting GAPDH) to impede the glycolytic escape route. Similarly, inhibiting branched-chain amino acid (BCAA) catabolism with gabapentin disrupts TCA cycle fueling, synergizing with copper to collapse mitochondrial metabolism [312].

Synergy with Immunotherapy: Cuproptosis is intrinsically immunogenic. It promotes mitochondrial DNA release, activating the cGAS-STING pathway and type I interferon response, enhancing dendritic cell maturation and CD8⁺ T-cell infiltration [313, 314]. This creates a rationale for combining cuproptosis inducers with immune checkpoint inhibitors (e.g., anti-PD-1/PD-L1 antibodies), potentially converting "cold" tumors into "hot" ones. Notably, DSF/Cu has been shown to enhance the efficacy of anti-PD-1 therapy in preclinical HCC models [315].

Multifunctional Nanoplatforms: Nanotechnology enables tumor-targeted delivery and combinational therapy in a single agent. Copper-based nanomaterials (e.g., metal–organic frameworks, mesoporous silica nanoparticles) [316–318] can co-deliver copper ions and other drugs (chemotherapeutics, metabolic inhibitors), respond to the tumor microenvironment (low pH, high GSH), deplete protective antioxidants like glutathione, and even mediate photothermal therapy [319, 320]. These platforms maximize intratumoral copper accumulation while minimizing systemic toxicity, effectively overcoming physiological barriers to cuproptosis induction.

Overcoming Therapeutic Resistance: Emerging strategies target specific resistance mechanisms. For example, in hypoxic tumors where HIF-1α upregulates copper-chelating metallothioneins, combining copper ionophores with HIF-1α inhibitors can resensitize cells [297, 321]. Furthermore, synthetic lethality approaches exploit cancer-specific vulnerabilities; ARID1A-deficient HCC cells, due to their heightened TCA cycle dependence [322, 323], show exquisite sensitivity to copper overload, pointing to biomarker-driven patient stratification.

The translational landscape of copper-targeted therapies is rapidly evolving, moving from broad depletion to precise induction of cell death and restoration of genetic function. The integration of these strategies with immunotherapy and nanotechnology holds exceptional promise for developing the next generation of targeted and effective treatments for liver diseases, cancers, and beyond. Key preclinical and clinical findings are summarized in Table 2.

**Table 2:** Therapeutic strategies targeting copper homeostasis and cuproptosis: a summary of preclinical and clinical investigations

Strategy category	Agent/platform	Key preclinical animal model findings	Core mechanism/target	Clinical translation status
**Copper Ionophore Monotherapy**	**Elesclomol (ES)**	The ES-Cu complex induces mitochondrial oxidative stress and proteotoxic death, suppressing tumor growth in models including glioblastoma stem-like cells and hepatocellular carcinoma (HCC) [85, 115, 116]	Functions as a copper shuttle. Cu^2^⁺ is reduced to Cu⁺ by FDX1, triggering lipoylated TCA cycle protein aggregation and cuproptosis [9, 47].	**Completed Phase II/III trials** (e.g., in melanoma). Not widely approved due to variable efficacy; currently revisited as a research tool and combination therapy component (NCT00522834) [300].
	**Disulfiram/Copper (DSF/Cu)**	In models of HCC, nasopharyngeal carcinoma, etc., DSF/Cu inhibits tumor growth/metastasis, reverses chemoresistance, and induces immunogenic cell death, enhancing anti-PD-1 efficacy [98, 110, 307, 324].	1) Proteasome inhibition and ER stress; 2) ROS generation and oxidative damage; 3) Inhibition of pro-survival pathways (e.g., NF-κB) [91, 325].	**Clinical repurposing is ongoing** DSF is FDA-approved for alcohol use disorder **Multiple Phase I/II trials** evaluating the anticancer potential, often combined with chemotherapy to overcome resistance (NCT00742911, NCT04265274) [326].
**Combination Strategies**	**Copper Ionophore + HIF-1α Inhibitor**	In chemoresistant HCC models, hypoxia-induced HIF-1α stabilization upregulates copper-sequestering proteins (e.g., MT2A), conferring resistance. Combining a HIF-1α inhibitor restores sensitivity to copper ionophores [297].	Targets the hypoxic TME. Counters HIF-1α-mediated copper chelation and metabolic rewiring (PDK1/3-DLAT axis) to resensitize cells to cuproptosis	**Preclinical proof-of-concept**. Identifies a novel approach to overcome microenvironment-driven resistance. No registered clinical trials for this specific combination, though HIF-1α inhibitors are under investigation in other oncology contexts
	**Targeting Metabolic Vulnerabilities (e.g., ARID1A deficiency)**	In ARID1A-deficient HCC models, enhanced dependence on mitochondrial respiration and the TCA cycle create synthetic lethality with copper overload, selectively eradicating otherwise resistant cells [299].	Exploits cancer-specific metabolic dependencies (due to gene loss like ARID1A) for precise targeting via cuproptosis	**Preclinical proof-of-concept**. Provides a rationale for patient stratification based on biomarkers (e.g., ARID1A mutation) for precision copper-based therapy
**Multifunctional Copper-Based Nanoplatforms**	**Metal–Organic Frameworks & Other Nanosystems**	Various engineered copper-based nanoparticles (e.g., stimuli-responsive, co-delivery systems) show potent antitumor efficacy in mouse models of HCC, breast cancer, etc. They overcome multidrug resistance, alleviate hypoxia, deplete glutathione, and synergistically enhance cuproptosis [42, 101, 298, 301].	1) Efficient copper delivery induces canonical cuproptosis; 2) Glutathione depletion disrupts redox balance; 3) ROS generation; 4) Combination with other modalities (e.g., chemotherapy, starvation therapy) [199, 203].	**Active area of preclinical development**. Most studies are in the animal model stage, representing a cutting-edge translational frontier. Advantages include multifunctionality, but challenges remain in manufacturing, pharmacokinetics, and safety profiling

## Perspective

The intricate balance of copper homeostasis and its recent connection to a novel regulated cell death pathway—cuproptosis—have placed copper biology at the forefront of research in metabolic diseases and oncology. This review systematically examines this evolving field, spanning fundamental physiology to therapeutic translation. Looking forward, the insights obtained highlight several sequential and compelling directions for future investigation.

Firstly, in systemic and cellular copper homeostasis, although the roles of transporters (e.g., CTR1, ATP7A/B) and chaperones (e.g., ATOX1, CCS, COX17) are well established, the regulatory mechanisms that fine-tune their activity in response to metabolic or oncogenic signals remain incompletely elucidated. Future studies should employ spatial transcriptomics and high-resolution live-cell imaging to visualize real-time copper flux and protein trafficking within specific subcellular compartments. Moreover, the newly identified role of ZnT1 in copper import underscores the potential for extensive interconnections with other metal homeostasis networks—an area ripe for exploration.

Secondly, the molecular mechanism of cuproptosis has been elegantly delineated, focusing on FDX1-mediated copper reduction and its binding to lipoylated TCA cycle proteins such as DLAT. Nevertheless, key questions remain: What are the precise structural determinants underlying copper-induced protein aggregation? How is the immunogenic cell death (ICD) signature initiated via mtDNA–cGAS–STING signaling precisely regulated, and how can it be harnessed without inducing counterproductive PD-L1 upregulation? A deeper investigation into these mechanisms—using cryo-EM to characterize aggregating complexes and genetic screens to identify novel regulators of downstream signaling—will be essential.

Thirdly, the critical interplay between copper homeostasis and cuproptosis underscores the cellular metabolic state as a decisive factor. The dependence on oxidative phosphorylation (OXPHOS) and the resistance conferred by glycolytic shifts (e.g., via CDKN2A) reveal a dynamic metabolic interplay. Future efforts should aim to map the “cuproptosis sensitivity landscape” across diverse cancer types and patient subtypes. This entails systematically examining how other metabolic pathways—such as branched-chain amino acid catabolism or glutaminolysis—intersect with copper toxicity. The objective is to develop predictive biomarkers, likely integrating metabolic enzyme expression, FDX1 activity, and copper transporter status, to identify patients most likely to respond.

Finally, these foundational insights are already driving therapeutic innovation. Next-generation strategies should extend beyond first-generation copper ionophores (e.g., elesclomol) and complexes (e.g., DSF/Cu). Rational design of tumor-specific nanodelivery systems responsive to the tumor microenvironment (e.g., low pH, high glutathione) is crucial to enhance efficacy and minimize systemic toxicity. Furthermore, intelligent combination therapies—pairing cuproptosis inducers with OXPHOS inhibitors, glycolytic blockers, or immune checkpoint inhibitors—represent the most promising clinical avenue. Translational studies should prioritize validating these combinations in physiologically relevant models, including patient-derived organoids and humanized mouse models, to rapidly bridge the gap between the bench and the bedside.

In summary, the journey from copper homeostasis to cuproptosis has introduced a new mechanistic framework for understanding cell fate and a promising therapeutic axis. By deepening molecular insights and strategically targeting metabolic dependencies and immune interactions, this field is well positioned to harness copper’s lethal potential for transformative treatments in liver diseases, malignancies, and other copper-related diseases.

## Data Availability

Not applicable.
